# Pollen-Modified Flat Silk Cocoon Pressure Sensors for Wearable Applications

**DOI:** 10.3390/s24144698

**Published:** 2024-07-19

**Authors:** Shengnan Wang, Yujia Wang, Yi Wang, Jiaqi Liu, Fan Liu, Fangyin Dai, Jiashen Li, Zhi Li

**Affiliations:** 1 State Key Laboratory of Resource Insects, Southwest University, Chongqing 400715, China; 2 Chongqing Engineering Research Center of Biomaterial Fiber and Modern Textile, College of Sericulture, Textile and Biomass Science, Southwest University, Chongqing 400715, China; 3 Key Laboratory of Sericultural Biology and Genetic Breeding, Ministry of Agriculture and Rural Affairs, Southwest University, Chongqing 400715, China; 4 Department of Materials, The University of Manchester, Manchester M13 9PL, UK

**Keywords:** microstructure, flat silk cocoon, pressure sensor, PPy

## Abstract

Microstructures have been proved as crucial factors for the sensing performance of flexible pressure sensors. In this study, polypyrrole (PPy)/sunflower pollen (SFP) (P/SFP) was prepared via the in situ growth of PPy on the surface of degreased SFP with a sea urchin-like microstructure; then, these P/SFP microspheres were sprayed onto a flat silk cocoon (FSC) to prepare a sensing layer P/SFP-FSC. PPy-FSC (P-FSC) was prepared as an electrode layer through the in situ polymerization of PPy on the FSC surface. The sensing layer P/SFP-FSC was placed between two P-FSC electrode layers to assemble a P/SFP-FSC pressure sensor together with a fork finger electrode. With 6 mg/cm^2^ of optimized sprayed P/SFP microspheres, the prepared flexible pressure sensor has a sensitivity of up to 0.128 KPa^−1^ in the range of 0–13.18 KPa and up to 0.13 KPa^−1^ in the range of 13.18–30.65 KPa, a fast response/recovery time (90 ms/80 ms), and a minimum detection limit as low as 40 Pa. This fabricated flexible P/SFP-FSC sensor can monitor human motion and can also be used for the encrypted transmission of important information via Morse code. In conclusion, the developed flexible P/SFP-FSC pressure sensor based on microstructure modification in this study shows good application prospects in the field of human–computer interaction and wearable electronic devices.

## 1. Introduction

With the rapid development of science and technology, people aspire to build an intelligent society in order to easily connect human beings with the material world around them, in which flexible sensors play a crucial role. With a flexible substrate as the supporting material, flexible pressure sensors can adapt to a variety of irregular surfaces, making up for the shortcomings of traditional rigid sensors while possessing good sensing performance; therefore, they are widely used in electronic skin [1], human–computer interaction [2], and smart medicine [3]. According to the sensing principles, flexible pressure sensors can be classified into piezoresistive, capacitive, piezoelectric, and triboelectric sensors [4]. Among them, piezoresistive pressure sensors have received widespread attention because of their simpler preparation process, lower cost and wide application.

Sensing performance, an important index to evaluate flexible pressure sensors, is closely related to the three main factors of microstructure, conductive materials, and substrates. Microstructures such as the pyramid structure [5,6,7], microsphere structure [8,9,10,11], porous structure [12,13,14,15], sea urchin-like structure [16,17,18,19,20,21], etc., have been proven to improve the sensing performance of pressure sensors. Inspired by the multilayer corrugated cardboard structure, Xu et al. [9] prepared a flexible pressure sensor using the conductive microspheres SiO_2_/polyaniline (PANI) (PS). These conductive PS microspheres distributed in the multilayer structure can acutely sense external pressure so that the prepared flexible pressure sensor exhibits an ultra-low detection limit of 10 Pa. Lu et al. [22] developed a flexible piezoresistive sensor with a hierarchical interlocked spinosum microstructure via laser direct writing technology, and this sensor exhibited an ultra-high sensitivity of 68.3 kPa^−1^. These sensors with elaborate microstructures significantly enhance the sensing performance. However, to simplify the processes for creating microstructures and to avoid a reliance on the involvement of sophisticated equipment in these processes, some materials with native structures that facilitate the binding of conductive materials and can provide a reasonable range of deformation have been introduced for developing flexible sensors. Sunflower pollen (SFP), with a hollow sphere and numerous surface micro-thorns, can withstand large mechanical deformations, and its special structures and properties can be used for the design and application of piezoresistive sensors. Conductive materials are mainly used to construct conductive networks on insulating substrates, and their structures and properties can affect not only the microstructure but also the performance of the prepared piezoresistive sensors. Commonly used conductive materials include carbon materials [23,24,25,26,27], MXene [28,29,30], conductive metal nanomaterials [31,32], and conductive polymers [33,34,35]. The conductivity, structure characteristics, and material cost are generally considered in order to select the appropriate conductive materials. Polypyrrole (PPy) is a positively charged conductive polymer [36] with excellent conductive properties, and its unique in situ polymerization from pyrrole (Py) ensures its firm coating on the surface of a flexible substrate [37], forming a conductive layer, which is favorable for developing flexible sensors. Xia et al. [38] prepared a piezoresistive sensor via the in situ polymerization of a MXene/PPy composite on a polydimethylsiloxane sponge, utilizing the synergistic effect between PPy and MXene; this fabricated sensor achieved a high sensitivity of 6.8925 kPa^−1^. The flexible substrate significantly affects the softness and comfort of the fabricated piezoresistive sensor, which determines its wearability. Textiles with porous and soft, breathable properties, are the ideal substrate for flexible sensors. For silk fabric, in addition to these excellent properties, the rich chemical groups on the silk fiber surface facilitate the binding of conductive materials during the preparation process. However, the woven silk fabric has to experience cocoon cooking, silk reeling, and spinning and weaving, which is complex and time-consuming. By changing the path from a three-dimensional (3D) scaffold to a two-dimensional (2D) plane during the spinning process of *Bombyx mori*, a Flat Silk Cocoon (FSC) with a natural non-woven structure can be prepared. The silk fibers randomly arrange on the FSC to ensure porous and breathable properties. Like a natural cocoon, an FSC has superior flexibility as well as better biocompatibility. Moreover, the spinning process can be controlled by adjusting the spinning time or the number of *Bombyx mori* on the plane to obtain an FSC with different sizes and layers. The silk sericin produced by *Bombyx mori* during the spinning process is a good natural adhesive for bonding silk fibers and neighboring FSC layers. Therefore, this controllable spinning process and the natural glue sericin of FSC are helpful for introducing and bonding conductive materials, contributing to fabricating the flexible sensors with a designed structure and desirable performance. In our previous study [39], a chitosan quaternary ammonium salt (HACC)/MXene/SFP (HMSFP) with a unique microstructure was prepared and then sprayed on the FSC to prepare a pressure sensor. Although this fabricated HMSFP-FSC pressure sensor exhibited excellent sensitivity and cyclic stability, the response/recovery time of the HMSFP-FSC pressure sensor was slow. The slow response/recovery time of the HMSFP-FSC pressure sensor can be attributed to the long distance from the transmission of the pressure signal to the conductive network. 

Here, in this study, FSC was used as a flexible substrate, and P-FSC was prepared as an electrode layer via the in situ polymerization of PPy on its surface, while P/SFP was prepared via the in situ growth of PPy on the surface of degreased SFP with an sea urchin-like microstructure; then, these P/SFP microspheres were sprayed onto the SFC to prepare a sensing layer. These layers were assembled to form a piezoresistive sensor P/SFP-FSC together with an interdigital electrode. The amount of sprayed P/SFP was optimized to achieve the best sensing performance of this P/SFP-FSC pressure sensor; its applications in the fields of micro-pressure monitoring, human body movement monitoring, and the encrypted information transmission of this fabricated sensor were investigated.

## 2. Experimental Section

### 2.1. Materials

*Bombyx mori* was provided by State Key Laboratory of Resource Insects of Southwest University (China). Pyrrole (C_4_H_5_N, Py) was provided by Shanghai Macklin Biochemical Technology Co., Ltd. (Shanghai, China). Iron (III) trichloride hexahydrate (FeCl_3_•6H_2_O) was provided by Shanghai Aladdin Biochemical Technology Co., Ltd. (Shanghai, China). Sunflower pollen was purchased from Alibaba (Inner Mongolia, China), with a particle size of about 20–30 microns. Polyimide (PI) interdigitated electrodes (10 mm × 20 mm, line width 200 μm, line spacing 200 μm) were purchased from Shenzhen Taobo Laser Application Technology Co., Ltd. (Shenzhen, China).

### 2.2. Preparation of PPy-Modified FSC (P-FSC)

**Figure 1:**
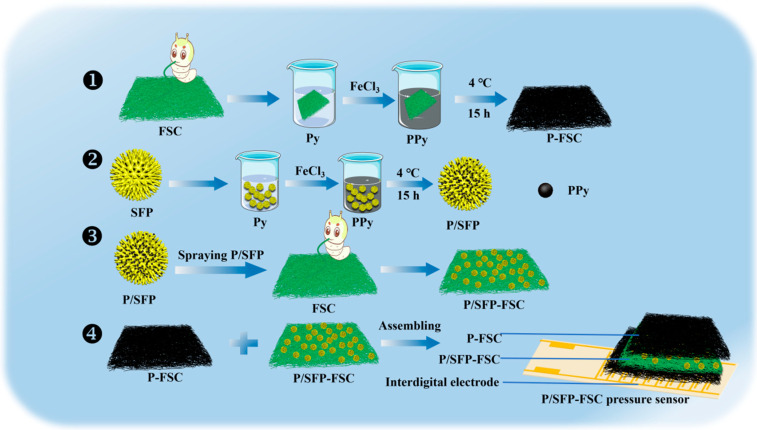
Flow chart of preparing P/SFP-FSC pressure sensor.

The *Bombyx mori* FSCs were made by irregularly swinging the *Bombyx mori* back and forth, allowing them to spit on a 2D plane; the silk was then directly impregnated in the pyrrole (Py) monomer solution for the in situ polymerization of PPy at a low temperature of 4 °C for 15 h. Ferric chloride (FeCl_3_) was used as the polymerization initiator in the Py monomer solution. The surface of the prepared P-SFC was rinsed with deionized water to remove the residual Fe^3+^ and the unpolymerized Py monomer, and then was dried for the electrode application (Figure 1).

### 2.3. Preparation of PPy/SFP-Modified FSC (P/SFP-FSC)

The defatted SFP was impregnated in Py monomer solution for in situ polymerization of PPy on the surface under the same condition described in 2.2 to prepare P/SFP, and then the prepared P/SFP was directly sprayed onto the surface of FSC to obtain the P/SFP-FSC as a sensing layer. The spraying amount per unit area of P/SFP was optimized and 2 mg/cm^2^, 6 mg/cm^2^, and 10 mg/cm^2^ were used, respectively (Figure 1).

### 2.4. Preparation of P/SFP-FSC Pressure Sensors

The prepared P/SFP-FSC was placed in the middle of two P-FSC layers and assembled face-to-face with the fork finger electrodes together, and then these assemblies were encapsulated with polyimide (PI) tape to fabricate the P/SFP-FSC flexible pressure sensor (Figure 1).

### 2.5. Characterizations

The micromorphology of the prepared samples was observed using scanning electron microscopy (SEM) (ZEISS Gemini 300, Carl Zeiss AG, Obercohen, Germany, 3 kV power). The elemental content and distribution were measured by using an energy dispersive spectroscopy (EDS) (OXFORD Xplore, Oxford Instruments Technology (Shanghai) Co., Ltd., Shanghai, China, 15 kV power). The sensing performance of the prepared sensors was tested by using a test system consisting of an electrochemical workstation (CHI660E instrument, Shanghai Chenhua Instrument Co., Ltd., Shanghai, China) and an electronic universal testing machine (E44.104, MTS Systems (Tianjin, China) Co. Ltd.).

## 3. Results and Discussion

### 3.1. Characterization of P/SFP-FSC Pressure Sensors

**Figure 2:**
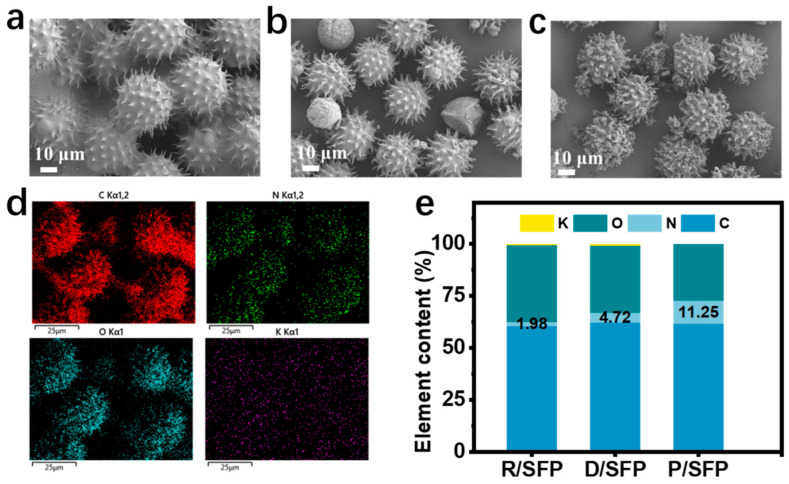
(**a**–**c**) SEM images of R/SFP, D/SFP, and P/SFP, respectively; (**d**) EDS mapping of P/SFP; (**e**) elemental content statistics of P/SFP.

We used the SEM and EDS images to analyze the morphology of SFP in different states; these images are shown in Figure 2. Figure 2a–c show the SEM images of pristine SFP (R/SFP), degreased SFP (D/SFP), and PPy-modified SFP (P/SFP), respectively. It can be clearly seen through the SEM images that the surface of the R/SFP is covered with a film-like layer consisting of beeswax, ash, and other impurities, which encapsulate the microspines on the surface of the SFP. After ultrasound repeated rinsing, the microspines on the SFP surface are clearly exposed with the removal of these substances, which ensures enough space for loading PPy on the surface. After in situ polymerization, the grown PPy is filled in the space among the microspines on the SFP surface, and this rough surface increases the specific surface area, in favor of capturing small force signals in sensing applications.

By scanning the elements on the surface of P/SFP (Figure 2d), it is observed that the C, N, and O elements are uniformly distributed on the surface of P/SFP, wrapping around the sea urchin-like microspheres, whereas the C and O elements mainly originate from the SFP and the N elements mainly originate from PPy. The statistical analysis of the EDS elemental content in Figure 2e reveals that the percentage of N elemental content for P/SFP increases significantly to 11.25% compared with R/SFP and D/SFP, indicating that the PPy is grown on the surface of P/SFP through in situ polymerization, which is the supplementary proof for the SEM images.

**Figure 3:**
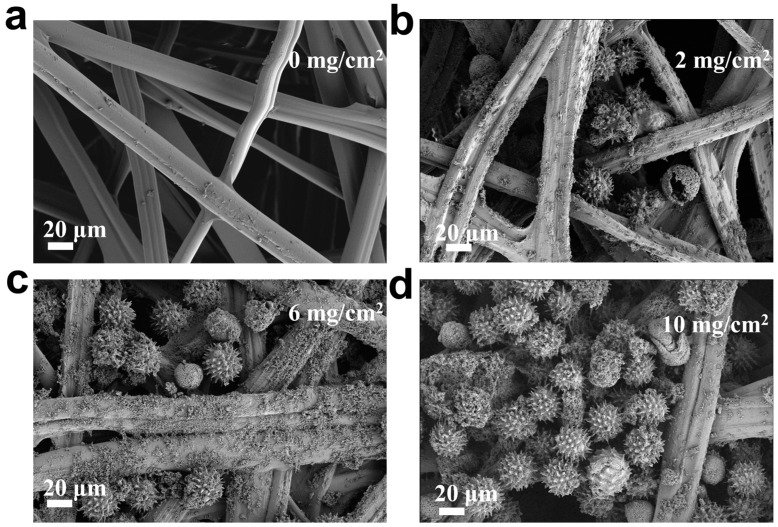
(**a**–**d**) SEM images of FSC surfaces sprayed with PPy/SFP at 0, 2, 6, and 10 mg/cm^2^, respectively.

In order to explore the optimal conditions for fabricating a flexible pressure sensor and to prepare the pressure sensor with excellent performance, the P/SFP microspheres were sprayed on the SFC with parameters of 2 mg/cm^2^, 6 mg/cm^2^, and 10 mg/cm^2^, respectively, and the surface morphologies of these prepared P/SFP-SFCs were observed by using SEM, as shown in Figure 3. As can be seen from the SEM images, the pristine FSC has smooth fibers and, among them, there is a plenty of space (Figure 3a). After 2 mg/cm^2^ of P/SFP was sprayed on (Figure 3b), the P/SFP microspheres with a sea urchin-like structure fill in the internal space of the FSC; also, the surface of the silk fibers is covered with a portion of the particles, which are probably the unloaded PPy particles. With an increase in the sprayed P/SFP amount to 6 mg/cm^2^ (Figure 3c) and 10 mg/cm^2^ (Figure 3d), more PPy particles are covered on the silk fibers and more P/SFP composites are filled in the space among the fibers of FSC. These P/SFP composites are packed tightly to each other, further narrowing the space, which probably changes the electronic transmission path and affects the sensing performance of the prepared sensor. 

### 3.2. Analysis of Sensing Performance and Sensing Principle of Pressure Sensors

**Figure 4:**
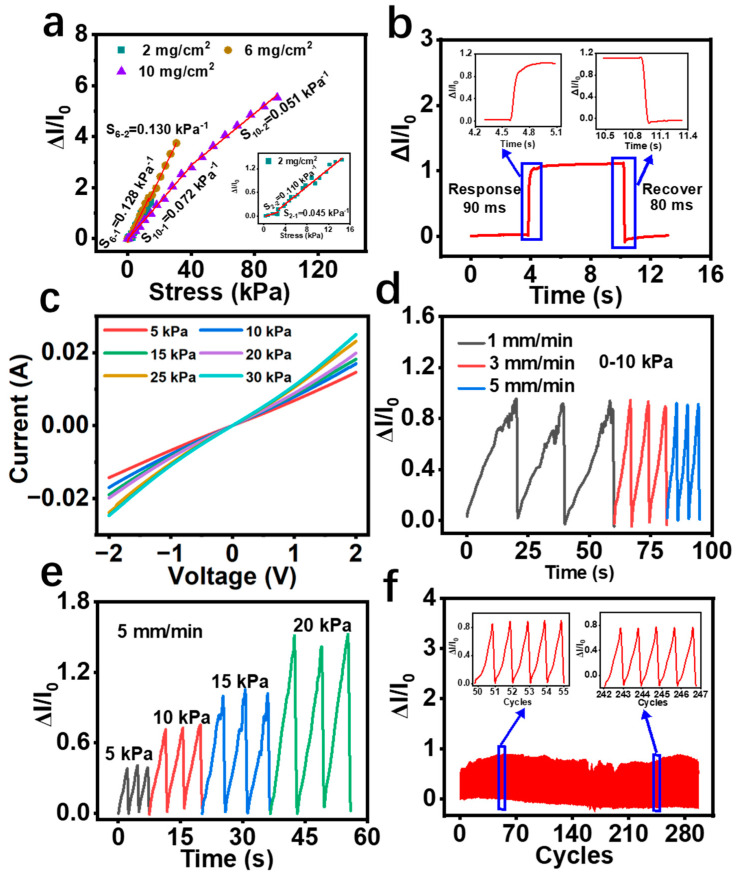
(**a**) Comparison of PPy/SFP−FSC pressure sensor sensitivity for different spray volumes per unit area; (**b**) response/recovery time; (**c**) I−V curves; (**d**,**e**) continuous dynamic response of the sensors for different loading rates and different loading pressures, respectively; (**f**) cyclic durability of the sensors.

**Figure 5:**
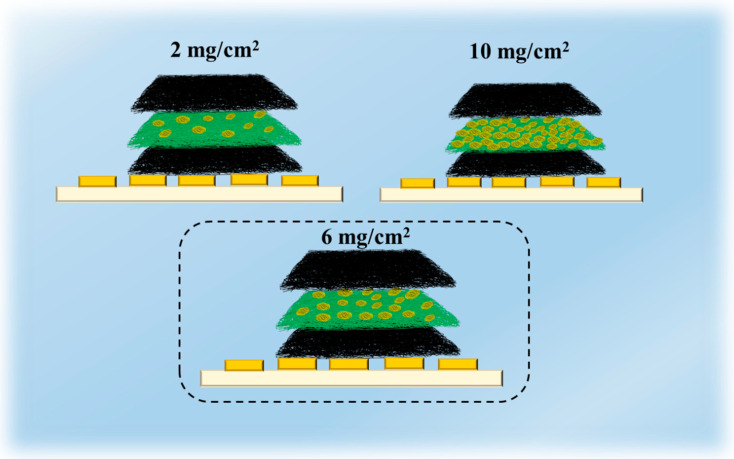
Schematic diagram of the sensing principle of the P/SFP-FSC pressure sensor.

The sensitivities of the prepared flexible pressure sensors with different spraying amounts per unit area of P/SFP microspheres were tested and shown in Figure 4a; these are represented as the slopes of the curves in this figure. The calculated sensitivities of these sensors and their pressure detection ranges are shown in Table 1, in which the pressure sensor with 6 mg/cm^2^ microspheres exhibits the highest sensitivity, 0.128 KPa^−1^ in the range 0–13.18 KPa and 0.13 KPa^−1^ in the range 13.18–30.65 KPa, while the sensors with less microspheres or more microspheres have the lower sensitivities. The number of changeable conductive paths within the P/SFP-FSC pressure sensors is the possible reason for this. As shown in Figure 5, more sprayed P/SFP microspheres within the sensor (10 mg/cm^2^) means more initial conductive paths being established, which in turn leads to a larger initial current of this pressure sensor. In this case, when pressure is applied to the pressure sensor, only a few conductive paths within the sensor are varied; thus, the current shows very small changes even when high pressure is applied. In contrast, when too few (e.g., 2 mg/cm^2^) sprayed microspheres are distributed within the sensor, they cannot ensure smooth connections among the silk fibers in the sensor. Even if the pressure is applied, the increased conductive paths are limited, which leads to an insensitive response to the pressure. It is proven that 6 mg/cm^2^ of sprayed microspheres is appropriate for fabricating the sensor with the best sensitivity, in which the number of initial conductive paths are within a reasonable range, ensuring that there is enough deformable space to cope with the pressure changes. Therefore, the P/SFP-FSC sensor with 6 mg/cm^2^ of P/SFP sprayed microspheres was applied to the following studies.

The response/recovery time of the fabricated P/SFP-FSC pressure sensor is shown in Figure 4b, where the response/recovery time of the sensor is 90 ms/80 ms, respectively, proving the fast response of this sensor to the external pressure changes. Under the applied voltage from −2 V to 2 V, with the increase in the applied pressure from 5 kPa to 30 kPa, the maximum current value of the curves also increases (Figure 4c), and all I-V curves under these applied pressure states pass the origin, proving that this prepared P/SFP-FSC flexible pressure sensor has good piezoresistive characteristics [40]. The frequency dependence can also reflect the performance of the sensor. Figure 4d shows that at different compression rates of 1 mm/min, 3 mm/min, and 5 mm/min, a pressure of 0–10 kPa is continuously and repeatedly applied to the surface of the sensor, and the displayed peak values of ΔI/I_0_ output from the sensor are the same, which proves that the sensor has a stable response at different compression rates and it does not have frequency dependence. Under a continuous and dynamically varying pressure applied to the sensor at a compression rate of 5 mm/min, the output ΔI/I_0_ value increases in a stepwise manner (Figure 4e), which proves the good dynamic pressure response of this sensor. In addition, a cyclic pressure of 0–10 kPa was continuously and repeatedly applied to the sensor at a compression rate of 5 mm/min, and the test results show that the prepared pressure sensor can withstand more than 300 times of cyclic loading/unloading pressure (Figure 4f), demonstrating that the prepared flexible pressure sensor P/SFP-FSC has a good cyclic stability that can ensure its service life. The P/SFP-FSC flexible pressure sensor proposed in this study exhibits good comprehensive performance in terms of sensitivity, response time, and detection range, compared with the reported similar flexible pressure sensors [9,39,41,42,43,44] (Table 2).

According to Figure 5, a flexible pressure sensor with good sensing performance and a sprayed number of P/SFP microspheres with a unit area of 6 mg/cm^2^ are analyzed for the change in the internal mechanism when subjected to external pressure. As can be seen in Figure 5, the sensor establishes a conductive pathway path that is moderate, allowing it to be recognized in the low- to high-pressure range. This is because the 6 mg/cm^2^ pressure sensor has more conductive pathways compared to the flexible pressure sensor with a P/SFP microsphere spray volume of 2 mg/cm^2^ per unit area, so it extends the detection range of the sensor. In contrast, for a flexible pressure sensor with a spray volume of P/SFP microspheres with a unit area of 10 mg/cm^2^, the pressure sensor with 6 mg/cm^2^ has a larger deformation space and thus an improved sensitivity. In addition, flexible pressure sensors with a sprayed number of P/SFP microspheres with a unit area of 6 mg/cm^2^ showed different sensitivities in low- and high-pressure environments, and the sensitivity in the high-pressure range was higher than that in the low-pressure range. This may be due to the microspine structure on the surface of the sprayed P/SFP microspheres that leads to a smaller change in sensitivity in the low-pressure range, where microsphere-to-microsphere contact has just begun and the change in contact area per unit is small. When in the high-pressure range, the microspheres are further contacted with each other, at which point there is a large variation in the contact area per unit, resulting in a large variation in its sensitivity. Therefore, the micro-spike structure on the surface of the P/SFP microspheres provides sufficient conductive paths and deformation space for the sensor to be sensitive to changes in external pressure.

### 3.3. Micro-Pressure Detection Performance Analysis

**Figure 6:**
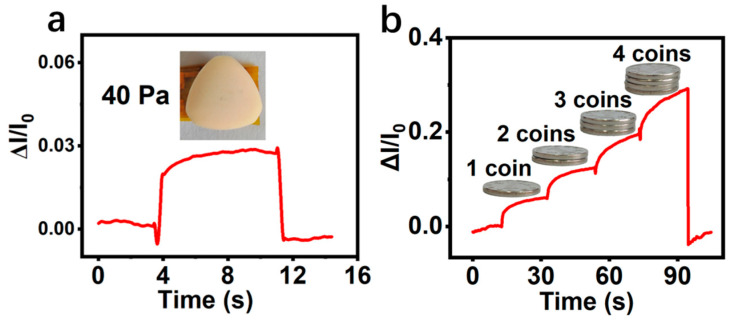
(**a**) Minimum detection limit of PPy/SFP-FSC pressure sensor; (**b**) response to successive coin increases.

Further, the minimum detection limit of the P/SFP-FSC flexible pressure sensor was investigated. The low pressure of about 40 Pa, which is comparable to the weight of a candy, was applied to the sensor surface and removed after 8 s, as shown in Figure 6a. The ΔI/I_0_ value shows a rapid increase at the moment of applying this low pressure, and then it returns to the initial state after the pressure is withdrawn, demonstrating that this sensor has an excellent response and recovery performance to pressures as low as 40 Pa. This sensitive response to a small pressure may be attributed to the micro-pricked convex structures on the surface of the P/SFP, which can acutely sense the subtle external pressure and enable the P/SFP-FSC pressure sensor to give a rapid response. This fabricated sensor can also monitor dynamic micro-pressure (Figure 6b). Four coins were successively placed on the surface of the sensor; accordingly, ΔI/I_0_ shows a step of successive increase, demonstrating the sensor’s ability to monitor dynamic micro-pressure.

### 3.4. Joint Movement Monitoring

**Figure 7:**
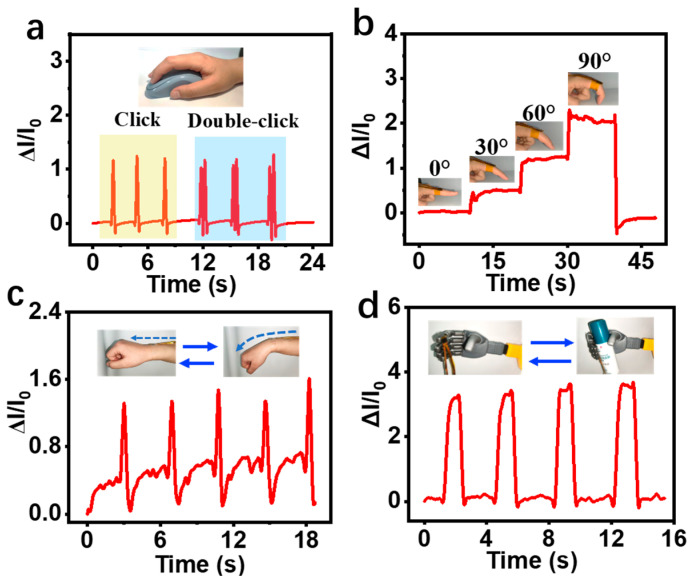
(**a**) Mouse click test; (**b**) finger bending test; (**c**) wrist bending test; (**d**) robotic arm grasping test.

Human motion monitoring is of great significance in the fields of human health, human–computer interaction technology, and smart medicine. Flexible sensors generally stick tightly to human skin, enabling better human motion detection. The prepared flexible PPy/SFP-FSC sensor was attached to the fingertip of the index finger, and then single-click and double-click operations for the mouse were carried out synchronously (Figure 7a); the output signal of the sensor presented a single peak and a double peak, indicating that this flexible sensor can clearly distinguish these different operations of the index finger. In addition, the prepared sensor was attached to a finger joint, and as the finger joint was bent to 30°, 60°, and 90°, the ΔI/I_0_ in Figure 7b increased step-by-step, corresponding to the degree of finger bending. Sticking the sensor to the wrist for monitoring also revealed that the sensor can stably sense and detect human motion through the output signal (Figure 7c), showing its ability to monitor large-scale joint activities. Furthermore, this P/SFP-FSC flexible pressure sensor was attached to the finger part of the robotic arm, simulating the grasping and placing of the bottle (Figure 7d). It is worth noting that during the process of grasping–placing, the output ΔI/I_0_ value of the sensor maintains a high degree of consistency and signal repeatability, which suggests that the sensor has great potential for future application in human–computer interaction.

### 3.5. Analysis of Results of Encrypted Message Transmission

**Figure 8:**
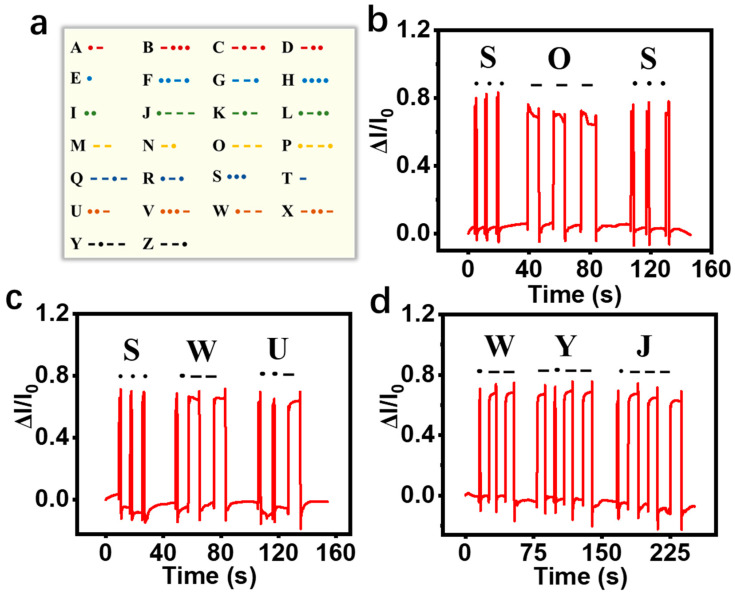
(**a**) Comparison table of Morse code; (**b**–**d**) response of the sensor when finger taps on different Morse codes ‘SOS’, ‘SWU’, and ‘WYJ’.

Morse code is a method of encrypting the transmission of information. As shown in Figure 8a, the 26 letters of the alphabet are combined based on a Morse code table to output information. As shown in Figure 8b–d, according to the Morse code table, tapping and combining ‘·’ and ‘-’ can output the signals representing ‘SOS’, ‘SWU’, and ‘WYJ’, indicating this sensor can be used to transmit encrypted information on special occasions or in disaster emergency rescue.

## 4. Conclusions

In this study, the P/SFP-FSC flexible pressure sensor was designed and developed by assembling a sea urchin-like PPy/SFP microspheres-modified sensing P/SFP-FSC layer, two PPy-FSC electrode layers and an interdigital electrode. This prepared pressure sensor exhibits an optimum sensitivity of 0.13 KPa^−1^ in the range of 13.18–30.65 KPa, response/recovery times of 90/80 ms, cyclic stability of more than 300 cycles, and a minimum pressure detection range of 40 Pa. This flexible P/SFP-FSC sensor can maintain a stable signal output under dynamically changing compression rates and pressures. With these favorable sensing properties, this sensor can be used for monitoring human motions and also can be used for the encrypted transmission of information in disaster emergency rescue. To sum up, the P/SFP-FSC flexible pressure sensor in this study shows great application potential in the field of human–computer interaction, intelligent medical care, and signal transmission.

## Figures and Tables

**Table 1:** Sensitivity and detection range of different samples.

Samples	Stress Range/kPa	Sensitivity/kPa^−1^
2 mg/cm^2^	0–2.72	0.045
	2.72–14.5	0.11
6 mg/cm^2^	0–13.18	0.128
	13.18–30.65	0.13
10 mg/cm^2^	0–40.14	0.072
	40.14–94.19	0.051

**Table 2:** Performance comparison between the P/SFP-FSC pressure sensor and the reported similar flexible pressure sensors.

Reference	Substrate Structure	Conductive Material	Sensitivity (kPa^−1^)	Response Time (ms)	Sensing Range (kPa)
[9]	SF/PLGA fibers	SiO_2_/PANI	0.071	145	0.01−380
[38]	flat silk cocoon	MXene	0.005	140	0–82
[40]	PLLA substrates	sliver paste	0.035	-	0–12
[41]	cotton fabric	LIG	0.0085/0.0012	70	0–30/30–220
[42]	silk fiber film	AgNWs	0.075/0.0084	-	0–10/10–53
[43]	nylon fabric	Ag	0.036	104	0.2–5
this work	flat silk cocoon	PPy/SFP	0.13	90	0–30.65

## Data Availability

The raw data supporting the conclusions of this article will be made available by the authors on request.
